# Sonication Leads to Clinically Relevant Changes in Treatment of Periprosthetic Hip or Knee Joint Infection

**DOI:** 10.7150/jbji.45006

**Published:** 2020-04-24

**Authors:** Marrit Hoekstra, Ewout S. Veltman, Ruben F.R.H.A Nurmohamed, Bruce van Dijk, Rob J. Rentenaar, H. Charles Vogely, Bart C.H. van der Wal

**Affiliations:** 1 Department of Orthopaedic Surgery, University Medical Centre Utrecht, the Netherlands; 2 Department of Clinical Microbiology, University Medical Centre Utrecht, the Netherlands

## Abstract

**Background:** Diagnosis of periprosthetic joint infection (PJI) can be troublesome. Sonication can be a helpful tool in culturing bacteria that are difficult to detect with standard tissue cultures. Aim of this study is to evaluate the clinical importance of our standardized sonication protocol in detecting periprosthetic joint infection.

**Materials and methods:** All patients with revision surgery of a hip or knee prosthesis between 2011 and 2016 were retrospectively reviewed and divided in two groups: clinically suspected of infection or not suspected of infection. For both tissue culture and implant sonication, calculations of sensitivity and specificity were performed. Clinical relevance of sonication was evaluated by calculating in which percentage of patients' sonication influenced clinical treatment.

**Results:** 226 patients with revision of a total hip prosthesis (122 patients) or a total knee prosthesis (104 patients) were included. Sensitivity of perioperatively taken tissue cultures was 94.3% and specificity was 99.3%. For sonication sensitivity was 80.5% and specificity was 97.8%.

In the infection group eight patients (9%) with only one positive tissue culture and a positive sonication fluid culture with the same pathogen were found.

**Interpretation:** Although sensitivity and specificity of sonication was lower compared to tissue cultures, periprosthetic joint infection could only be established in 8 patients (9%) suspected of infection because of a positive result of the sonication fluid culture.

Sonication leads to clinically relevant changes in treatment and seems therefore to be a helpful diagnostic tool in clinical practice.

## Introduction

Prosthetic joint infection is recognized as a serious complication following arthroplasty of the hip or knee. About 1-2% of primary arthroplasties become infected, an incidence that increases to 10% for revision arthroplasties 1. Worldwide the numbers of annually performed primary and revision arthroplasties are increasing 2. The rise in absolute number of arthroplasties will result in an absolute increase in the number of PJI, even when the incidence of PJI can be decreased.

Diagnosis of infection can be troublesome, especially in cases of low-grade chronic infection. The suspicion of infection is confirmed when cultures of synovial fluid and periprosthetic tissue turn positive 3,4. However, the sensitivity and specificity of standard tissue cultures are low, as they are reported to be 57-61% and 97-99% respectively 5,6. The low sensitivity of tissue cultures causes too many false negative outcomes, which could lead to underestimation of the number of infections.

Unrecognized and therefore untreated infections lead to impaired outcome for the patient 7. Sonication of the removed prosthetic materials has been advocated to improve the postoperative culture results 6,8-12. This was backed-up by the international consensus meetings in Philadelphia in 2013 and 2018, with the advice to use sonication as an adjunct to periprosthetic tissue cultures 3**.**

Currently, synovial fluid and periprosthetic tissue cultures are the standard diagnostic modalities for the detection of the micro-organism. Unfortunately there still is a high false negative rate. This may be due to the difficulty of detecting bacteria present in the biofilm. Microorganisms, especially the slow-growing and less virulent ones that are enclosed in the glycocalyx matrix are hard to detect. Sonication is thought to disrupt the biofilm, and can be used as an adjunct diagnostic tool.

A major challenge in the management of these infections is the identification of the causative pathogen, as Rothenberg and colleagues showed that the infection eradication rate is improved when the causative organism is known 5.

Aim of this study is to evaluate the clinical importance of our standardized sonication fluid culture protocol in detecting periprosthetic joint infection, with an emphasis on its clinical consequences on the treatment of patients.

## Materials and Methods

This study was approved by the local research board. The STROBE statement was consulted while constructing the study and writing the manuscript.

### Study population

We retrospectively reviewed all patients who underwent revision hip or knee joint arthroplasty at our clinic between 2011 and 2016 and of which prosthetic material was used for sonication. Accuracy of our database was assessed by review of records of sonication fluid cultures at the Department of Medical Microbiology that serves our orthopaedic clinic. Exclusion criteria were availability of less than five tissue cultures, or absence of sonication fluid culture. None of the patients had received antibiotics preoperatively.

Patients were classified as being infected and included in the PJI group, based on the PJI criteria as postulated by the International Consensus Meeting in 2013 13. These criteria state that PJI is present when one of the major criteria exists or three out of five minor criteria exist. Major criteria are two positive periprosthetic tissue cultures with phenotypically identical organisms or a sinus tract communicating with the joint. Minor criteria are 1 an elevated serum C-reactive protein (CRP) and erythrocyte sedimentation rate (ESR), 2 elevated synovial fluid white blood cell (WBC) count or a positive change on leukocyte esterase test strip, 3 elevated synovial fluid polymorphonuclear neutrophil percentage (PMN%), 4 positive histological analysis.

### Tissue cultures

Using our standard treatment protocol for infection revisions, at least five cultures were taken during revision surgery from different locations in the hip or knee. Typically tissue samples were taken from the bone-prosthesis interfaces and from areas that were suspected of being infected. Synovial fluid was collected by puncture just before opening the joint capsule. All tissue cultures were taken with a different sterile rongeur forceps and placed in a separate sterile container. Antibiotic prophylaxis was withheld until all five cultures were collected. The culture materials were delivered to the microbiology laboratory immediately postoperatively for further processing.

### Microbiological procedures

Tissue specimens were homogenised using a bead-beater protocol. Homogenised tissue specimens were cultured on blood agar (BA, 4 days, aerobically), chocolat agar (GC, 3 days 5% CO_2_ ) and McConkey agar (McC) (2 days, aerobically) and Brucella blood agar (BBA, 14 days, anaerobically), as well as in Brain heart infusion broth, with added haemin and nicotinamide adenine dinucleotide (BHXV, 7 days aerobically). Pus samples were cultured on BA (4 days, aerobically), GC (3 days 5% CO_2_) McC (2 days, aerobically) and BBA (14 days, anaerobically).

### Sonication procedure

After explantation, the removed prosthetic components were placed in a sterile container. Ringers lactate was added by the surgeon until the prosthesis was covered for 90%. Prosthetic components were processed by the microbiology laboratory within 4 hours after removal. Upon arrival in the laboratory, the container was firmly shaken for 30 seconds. Then the container was placed in the sonication bath (Bandelin Bactosonic) for one minute on hundred percent power (200 W, power density ~ 0.22 W/cm^2^). Afterwards, the container was firmly shaken for 30 seconds again. One hundred microliters of uncentrifuged sonication fluid is cultured on BA and GC (5 days 5% CO_2_ ), on BBA (10 days, anaerobically) and in thioglycolate broth (10 days aerobically, followed by subculuture on BA (CO_2_) and BBA(anaerobically) for 4 days). Growth of different colonial morphologies was identified using MALDI-TOF MS (MBT Smart, research use only and security-related (SR) databases, Compass software, Bruker, Germany). Susceptibility testing was performed on isolates using Phoenix automated susceptibility testing (enterobacterales, staphylococci, enterococci) or disk diffusion and/or E-test (other isolates according to EUCAST methodology (disk diffusion) and manufacturer's instructions (Etest)). MIC values and disk diffusion growth inhibition zone diameters were interpreted according to EUCAST criteria.

Growth from sonication fluid, not deemed contaminants at the discretion of the attending clinical microbiologist, was reported quantitatively as colony-forming units per ml sonication fluid. Growth from thioglycolate broth only, was reported as sporadic growth without quantitation and, at least in case of anaerobes, were deemed clinically significant.

### Statistical analysis

Descriptive statistics were used to describe general patient characteristics. We used percentages for binary data, mean and/or standard deviation for logically distributed numerical data and median, range and/or percentiles for skewed data. Sensitivity and specificity for both tissue cultures and sonication fluid cultures were calculated using 2x2 contingency tables.

The statistical analysis was performed using SPSS version 22.0.

## Results

We identified 289 patients of interest from the two aforementioned databases. 63 patients were excluded using our exclusion criteria. A total of 226 patients treated with revision total hip or knee arthroplasty between 2011 and 2016 and at least five tissue cultures and sonication results available were included in the study. General patient characteristics were extracted from the charts and can be found in Table 1. Results of our study and a comparison with the literature search on sensitivity and specificity of tissue and sonication cultures can be found in Table 2 5,9,14-28.

In this study we found that the sensitivity of tissue cultures was 94.3% and the specificity was 99.3%. For sonication fluid cultures the sensitivity was 80,5% and the specificity was 97,8%.

In the infection group, we found eight patients (9%) with only one positive tissue culture and a positive sonication fluid culture with the same pathogen. The causative pathogens of the infections that were only confirmed by sonication fluid culture were *Streptococcus mitis* in two cases and *Staphylococcus epidermidis, Aggrigatibacter species, Cutibacterium acnes,* and* Corynebacterium striatum* in one case each. All other patients had multiple positive tissue cultures which were positive.

## Discussion

In this study we evaluated the use of our tissue and sonication fluid protocol used to diagnose PJI. Aim of the study was to investigate whether the use of sonication led to clinically important changes in the treatment of patients suspected of periprosthetic joint infection. Our results show that the sonication fluid culture changed the diagnosis to infection in eight of the eighty-seven patients with a PJI. Without sonication, these patients would have been undertreated for their infection as tissue cultures were unable to conclude the same.

Moreover we evaluated the sensitivity and specificity of our tissue and sonication fluid cultures. For tissue cultures and sonication fluid cultures we found a sensitivity of 94% and 81% and a specificity of 99% and 98% respectively. The sensitivity of our protocol was relatively high compared to earlier studies, as is shown in Table 2.

In our clinical practice sonication had additional value in 8 out of 87 patients (9%). The microorganisms that were solely identified by sonication were all low-virulent pathogens that produce biofilm. These infections are notoriously difficult to detect, as the preoperatively taken synovial fluid or tissue cultures are often false-negative. Jacobs et al showed that around 10% of aseptic revisions were found to have positive cultures, with a worse infection free survival compared to those with negative cultures. This could implicate that especially in a patient with a suspected early aseptic loosening of a hip or knee prosthesis and a negative preoperative synovial fluid culture, sonication should be performed to rule out infection, as tissue cultures may underestimate the number of infections. In recent years many advocates and opponents of the use of sonication fluid cultures have published their results (Table 2). Advocates of sonication mostly emphasize the increased specificity by combining tissue and sonication fluid cultures. Opponents of sonication mainly target the low sensitivity of sonication fluid cultures, as this limits its ability to exclude infection when the culture results return negative. Looking at the results of our study and the literature search in table 2 one might agree with the opponents of using sonication fluid culture as a diagnostic tool for confirming an infection, as the sensitivity of sonication fluid cultures is low and there are many false-negative results. However this does not take into account that in 9% of the patients in our study, the diagnosis of infection could only be established with the positive result of the sonication fluid culture. Sonication fluid cultures were essential to confirm the microbiological diagnosis of infection in a quite large proportion of patients in our study.

This study has several limitations, mostly reflected by the retrospective nature of the study. Determination of infection status was performed retrospectively using tissue culture results, therefore this results does not mimic the preoperative outpatient clinic setting. However, now we are sure that all patients in the infected group were actually infected.

However, this study is the first study to account the result that really matters to the patient, as we describe the number of patients for who the outcome of the sonication fluid culture was instrumental by detecting the infection and changing the treatment strategy. This may have prohibited persisting low-grade infection and subsequent early failure of the prosthesis in these selected patients.

Orthopaedic surgeons should be very reluctant to undertreat patients with a low-grade infection, as this may worsen the outcome of their patients 7. We advise orthopaedic clinics that treat patients with revision arthroplasty for suspected low-grade periprosthetic joint infection or aseptic loosening to perform sonication of the extracted prosthetic components, as the microbiological diagnosis of infection may otherwise be missed in about 10% of cases. Sonication of the explanted prosthetic components especially seems to have additional value in detection of low-virulent biofilm producing microorganisms that cause chronic periprosthetic joint infections.

However, sonication is not the only option for improved pathogen detection, new alternatives are being explored. Perhaps improving the current methods of tissue and synovial fluid cultures should be optimized. De Vecchi et al, for example, showed that dithiothreitol treatment for processing periprosthetic tissue showed higher sensitivity and specificity of detection of bacteria compared to routinely used methods 29. Both sonication and duthiothreitol are thought to be cost effective 27. Furthermore, Li et al show promising results of the diagnostic value of sonication fluid in blood culture bottles 30. Another possible alternative is next generation sequencing of synovial fluid. Tarabichi et al indicate that this method can identify periprosthetic infection in both culture positive as culture negative samples 31. Mariaux et al report that performing PCR on the sonication fluid of extracted material did not improve the bacterial detection and did not help to predict whether the patient will present a persistent or recurrent infection 32.

The diagnostic challenge is at hand for the treating orthopaedic surgeon and the orthopaedic community. Recently Parvizi and colleagues have adjusted and validated the infection criteria to improve the specificity and sensitivity to 99,5% and 97,7% respectively 33. It has yet to be studied whether these findings are reproducible in other settings. Until such answers are readily available, orthopaedic surgeons should use the resources available to confirm or refute the diagnosis of periprosthetic joint infection, especially in cases suspected of chronic low-grade infection.

## Figures and Tables

**Table 1:** General patient characteristics.

	PJI	Aseptic Failure
**Patients, n (%)**	87 (39)	139 (61)
**Female, n (%)**	46 (53)	75 (54)
**Mean age years (range)**	70 (40-92)	69 (35-92)
**Joint, hip (%)**	55 (63)	67 (48)
**Joint, knee (%)**	32 (37)	72 (52)

**Table 2:** Sensitivity and specificity of tissue and sonication cultures, compared to the literature.

Author	Tissue cultures		Sonication	
	Sensitivity (95% CI)	Specificity (95% CI)	Sensitivity (95% CI)	Specificity (95% CI)
**This study**	0.94 (0.87-0.98)	0.99 (0.96-0.99)	0.81 (0.71-0.88)	0.98 (0.94-0.99)
**Trampuz 2007**			0.61	0.99
**Kobayashi 2008**			0.86 (0.42-1.00)	0.84 (0.71-0.94)
**Piper 2009**			0.58 (0.39-0.75)	0.99 (0.95-1.00)
**Portillo 2012**			0.96 (0.79-1.00)	1.00 (0.94-1.00)
**Esteban 2012**			0.84 (0.66-0.95)	0.68 (0.52-0.81)
**Gomez 2012**			0.70 (0.62-0.78)	0.98 (0.95-0.99)
**Cazanave 2013**			0.77 (0.69-0.84)	0.98 (0.96-0.99)
**Ryu 2014**			0.78 (0.56-0.93)	1.00 (0.75-1.00)
**Rak 2016**			0.93 (0.77-0.99)	0.93 (0.83-0.98)
**Prieto Borja 2017**			0.62 (0.42-0.79)	0.97 (0.87-1.00)
**Van Diek 2017**	0.68 (0.56-0.78)	0.80 (0.74-0.86)	0.47 (0.35-0.59)	0.99 (0.96-1.00)
**Rothenberg 2017**	0.70 (0.58-0.80)	0.97 (0.81-1.00)	0.97 (0.89-0.99)	0.90 (0.72-0.97)
**Tani 2017**	0.56 (0.42-0.68)	0.94 (0.84-0.99)	0.77 (0.65-0.87)	0.98 (0.89-0.99)
**Renz 2017**	0.51 (0.40-0.63)	1.00 (0.89-1.00)	0.58 (0.46-0.69)	1.00 (0.89-1.00)
**Yan 2018**	0.66		0.73	
**Sambri 2018**	0.79 (0.69-0.87)	1.00 (0.98-1.00)	0.89 (0.75-0.96)	0.95 (0.87-0.99)
**Romano 2018**	0.71	0.76	0.71	0.94
**Renz 2018**	0.66	1.00	0.84	1.00
